# Death from human cytomegalovirus infection in a girl with congenital thymic dysplasia

**DOI:** 10.1186/s12985-022-01915-x

**Published:** 2022-11-08

**Authors:** Yang Liu, Yu Zhu, Weiping Liu, Chaomin Wan, Qin Guo

**Affiliations:** 1 Department of Pediatrics, West China Second Hospital, Sichuan University, No.20, 3Rd Section of Renmin South Road, Chengdu, 610041 People’s Republic of China; 2 Key Laboratory of Birth Defects and Related Diseases of Women and Children, Ministry of Education, Chengdu, People’s Republic of China; 3 NHC Key Laboratory of Chronobiology, Sichuan University, Chengdu, People’s Republic of China; 4 Department of Pathology, West China Hospital, Chengdu, People’s Republic of China

**Keywords:** Congenital thymic dysplasia, Cytomegalovirus, Refractory, Resistance

## Abstract

We report the case of a girl with congenital thymic dysplasia and refractory disseminated Human Cytomegalovirus (CMV) infection diagnosed by autopsy. Additionally, she was diagnosed with T-cell lymphopenia immunodeficiency and received antiviral therapy with ganciclovir (GCV) /valganciclovir (V-GCV) and enhanced foscarnet. The CMV viral load (VL) monitoring was elevated with retinitis, interstitial pneumonia, and hepatitis. The phenotype of T-cell lymphopenia was uncertain, which limited any alternative therapy by whole-exome sequencing (WES) and lymphocyte subset panel until autopsy. The girl died of progressive respiratory failure and septic shock at ten months of age. Severe disseminated CMV infection typically develops in infants with primary maternal infections and occurs earlier during gestation and in people with a weakened host immune system. Individuals with CMV infection with initial immunodeficiency are associated with a poor prognosis, which is similar to patients with secondary immunodeficiency. This case describes the difficult treatment and prognosis of CMV infection in patients with congenital immunodeficiency, highlighting the importance of early aggressive anti-CMV antiviral therapy in immunodeficiencies, VL monitoring, drug resistance and the role of T-cells in CMV infection.

## Background

The Human Cytomegalovirus (CMV) or human herpes virus-5 is a member of the family *Herpesviridae* [1]. It is a prevalent viral pathogen that causes primary infection, usually during childhood. CMV infection cannot be cleared from the host and produces a wide spectrum of diseases, ranging from asymptomatic infection to severe multiorgan systemic diseases [2, 3]. Congenital cytomegalovirus (cCMV) infection is a common congenital infection that may result in disrupted fetal development, intrauterine fetal demise, serious congenital malformations, and long-term neurodevelopmental sequelae, such as mental retardation and sensorineural hearing loss (SNHL); however, most neonates are asymptomatic [1]. CMV also can cause life-threatening diseases in immunocompromised patients, whether the infection is reactivated or acquired via primary CMV infection. Particularly, primary CMV infections or reactivation produce severe diseases in early-onset children with primary T-cell lymphopenia, nature killer (NK)-cell lymphopenia, and many combinations of immunological defects due to immune dysregulation and persistence of CMV infection related to T-cell exhaustion [4, 5]. Refractory or resistant CMV infection is also challenging due to the long-term use of limited antiviral drugs and an increased number of vulnerable patients [6]. While refractory CMV infection is defined as viremia that increases after at least two weeks of adequate antiviral therapy, antiviral drug resistance refers to a viral genetic alteration that decreases susceptibility to one or more antiviral drugs [7].

Children with severe combined immunodeficiency (SCID) with resultant severe T cell lymphopenia are at risk of severe CMV infection [3]. As a select group of patients, congenital thymic dysplasia represents the archetype of thymic changes in cellular immunodeficiency, leading to reduced or absent cellular immune reactivity with normal immunoglobulin levels [8]. However, there are few reports of congenital thymic dysplasia exacerbated by CMV infection in primary immunodeficiency with T-cell lymphopenia. Here, we present the case of a 10-month-old girl with congenital thymic dysplasia who had refractory disseminated CMV infection after the antiviral therapy failed.

### Case presentation

The girl was born to non-consanguineous parents at 39 weeks of gestation, with a birth weight of 3400 g and a normal head circumference of 34 cm. She had a brother who died of pneumonia at the age of five months. She had newborn pneumonia and jaundice, but her condition improved, and was discharged five days later after treatment with piperacillin/tazobactam.

**Fig. 1:**
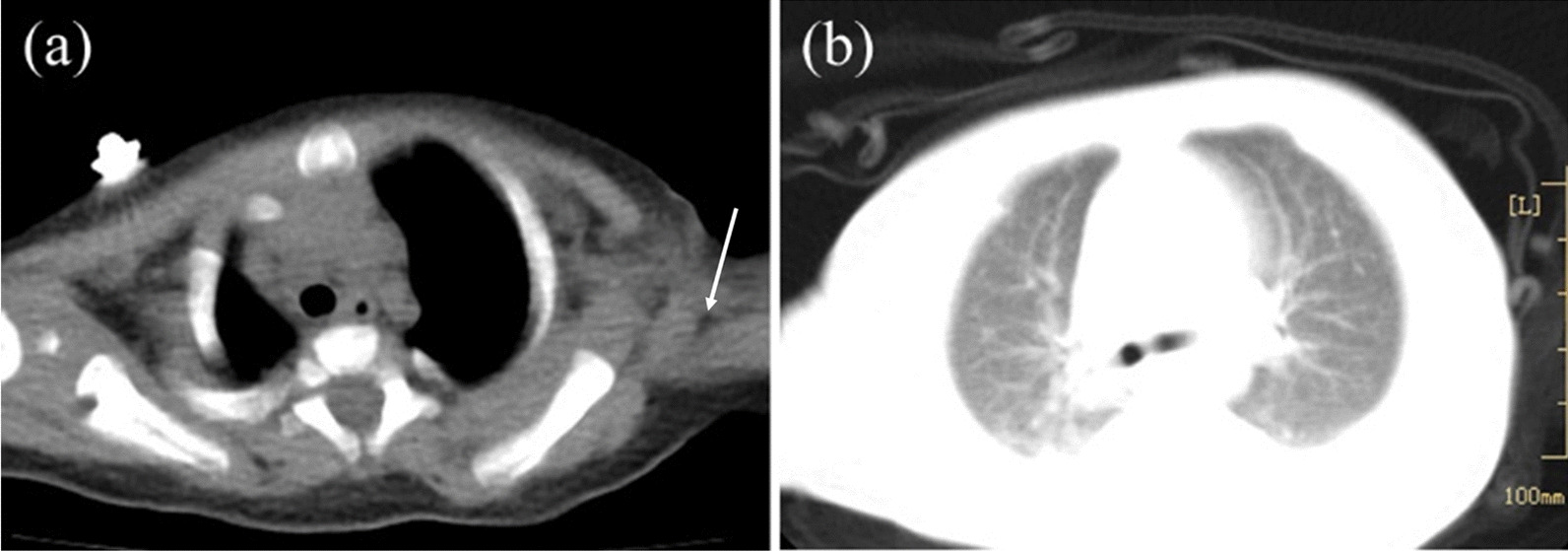
Thorax computed tomography (CT) was obtained at five months. **a** Left axillary lymph nodes (arrows). **b** Scattered patchy shadows in the lungs

**Fig. 2:**
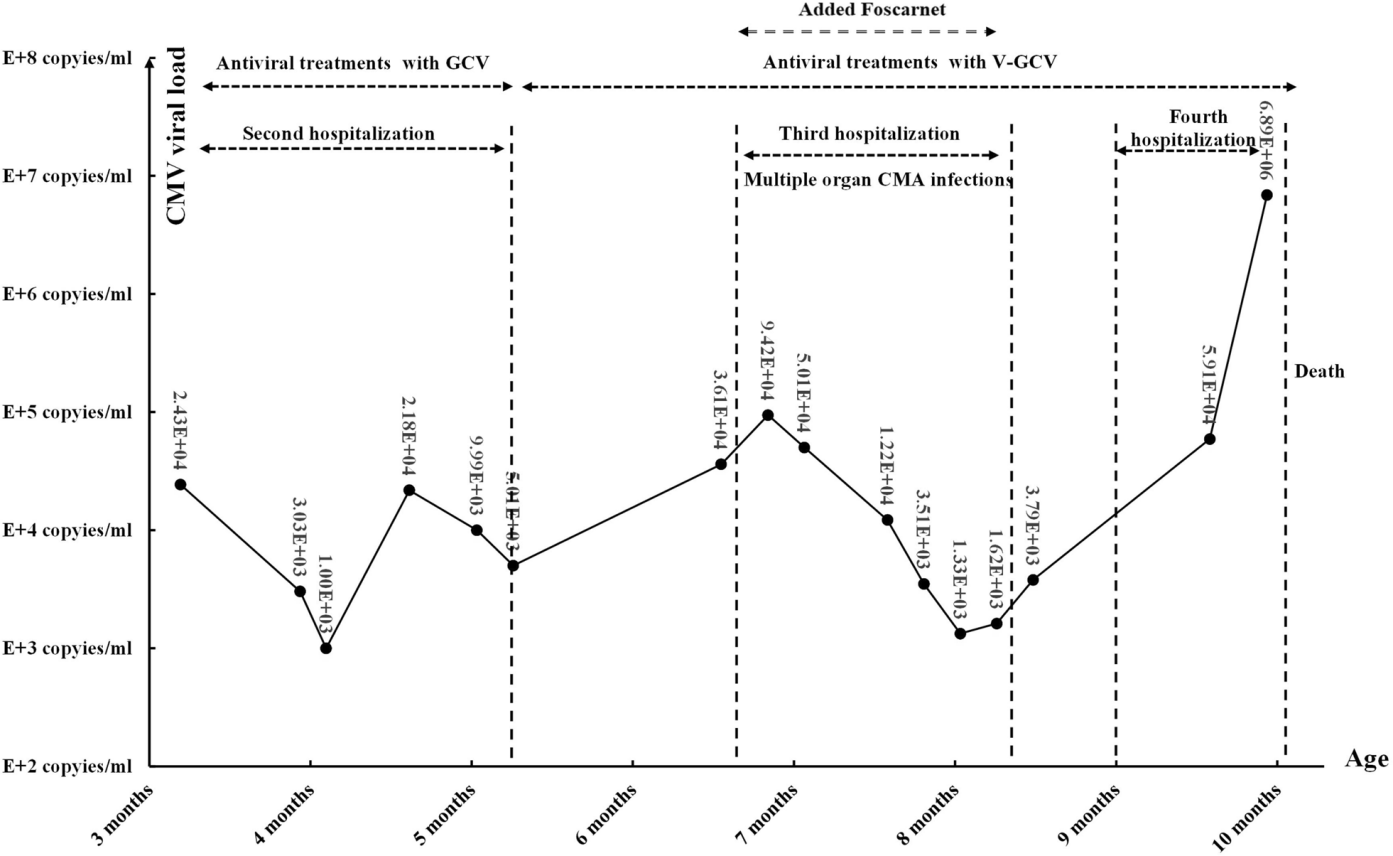
Changes in Cytomegalovirus (CMV) viral load (VL) and antiviral therapy

The patient presented dyspnea and tachypnea, and was admitted to the intensive care unit (ICU) at three months of age. The patient weighed 5 kg and appeared to be malnourished. During the physical examination, a sinus from the BCG vaccination scar measuring approximately 0.5 × 0.3 cm in the left upper arm was identified, and the secretion was Xpert/MTB (*Mycobacterium tuberculosis*) positive. Hepatomegaly was present (liver 4 cm below costal margin), and the laboratory tests revealed liver dysfunction, anemia, hypoproteinemia, and thrombocytopenia (Table 1). A computed tomography (CT) scan of the chest revealed swollen lymph nodes in the left axilla and patchy shadows in both lungs (Fig. 1). She was diagnosed with CMV infection and T-cell lymphopenia immunodeficiency with the following clinical history: (a) persistent respiratory tract infection; (b) presence of tuberculosis in a left axillary lymph node; (c) lymphocyte subset panel demonstrated the T-cell lymphopenia (Table 2); and (d) severe malnutrition. However, the etiology of her persistent immunodeficiency remained unknown. Serological markers for the human immunodeficiency virus (HIV) were negative. A virological test depicted a negative *CMV* immunoglobulin M (*CMV*- IgM) and positive *CMV* immunoglobulin G (*CMV*- IgG). The CMV VL measured with polymerase chain reaction (PCR) was very high (Fig. 2).

**Table 1:** Four Times of Hospitalization laboratory workup

	First hospitalization	Second hospitalization	Third hospitalization	Fourth hospitalization	Reference ranges
Age of months	Newborn	3	7	9	
*Hemogram*					
White blood cell count (/mm^3^)	10,000	9600	6600	18,400	4560–14,860
Hemoglobin level (g/L)	182	69	110	131	115–155
Platelet count (/mm^3^)	376,000	315,000	220	229	151,000–536,000
*Coagulation parameters*					
APTT (seconds)	–	36.2	36.6	81.9	18.8–38.8
PT (seconds)	–	11	10.5	14.1	8.7–14.7
Fibrinogen (mg/dL)	–	147	182	137	200–400
*Biochemistry panel*					
Alanine aminotransferase (U/L)	14	58	222	103	9–49
Aspartate aminotransferase (U/L)	51	132	384	225	14–40
Total bilirubin (μmol/L)	299.2	3.0	0.3	0.4	5–2.1
Direct bilirubin (μmol/L)	22.1	3.0	0.3	0.4	< 6.8
Total protein (g/L)	59	43.1	49.6	41.9	51–73
Albumin (g/L)	37.9	19.6	27.7	23.9	38–54
Gamma-glutamyl transferase (U/L)	96	20	64	13	12–38
Lactate dehydrogenase (U/L)	272	2859	1409	2098	120–246
BUN (μmol/L)	2.3	2.04	0.94	1.76	3.2–8.2
Creatinine (μmol/L)	27	15	13	12	17.3–54.6
*Inflammatory marker*					
C-reactive protein (mg/L)	< 0.5	12.4	< 0.8	16	0–8
Procalcitonin (ng/mL)	–	0.68	0.18	0.84	< 0.05

APTT activated partial thromboplastin time, PT prothrombin time, BUN blood urea nitrogen

**Table 2:** Immunological function tests

	First hospitalization	Second hospitalization	Third hospitalization	Fourth hospitalization	Reference ranges
Age of months	Newborn	3	7	9	
*Immunoglobulin levels*	–				
IgG, serum (g/L)	–	6.66	4.0	4.47	2.86–16.8
IgA, serum (g/L)	–	0.68	1.81	2.36	0.1–1.31
IgM, serum (g/L)	–	1.18	0.2	0.43	0.21–1.92
IgE, total (IU/mL)	–	9.98	27.7	26.6	< 165
*Lymphocyte subset panel*					
CD4 + lymphocyte (%)	–	11.0	3.6	2.6	33–58
CD4 + lymphocyte count (/mm^3^)	–	182	36	30	1500–5000
CD8 + lymphocyte (%)	–	2.9	3.4	1.6	11–25
CD8 + lymphocyte count (/mm^3^)	–	48	34	20	600–2000
NK cell (%)	–	18.7	25.3	2.1	2–14
NK cell count (/mm3)	–	310	258	30	100–1000

*Ig* immunoglobulin, *CD* cell differentiation, NK natural killer cell

The patient was intubated and ventilated artificially for respiratory distress. A standard INH-RIF-EMB-PZA anti-TB treatment regimen was provided. The patient was started on antiviral therapy with GCV (5 mg/kg, twice a day) and antibiotic therapy with vancomycin, meropenem, and cefoperazone/sulbactam. She was discharged with oral V-GCV (16 mg/kg, twice daily) from our hospital on the 50th day of admission with an improved condition, and the level of CMV VL decreased to 5010 copies/mL in the blood (Fig. 2) when she was five months old.

**Fig. 3:**
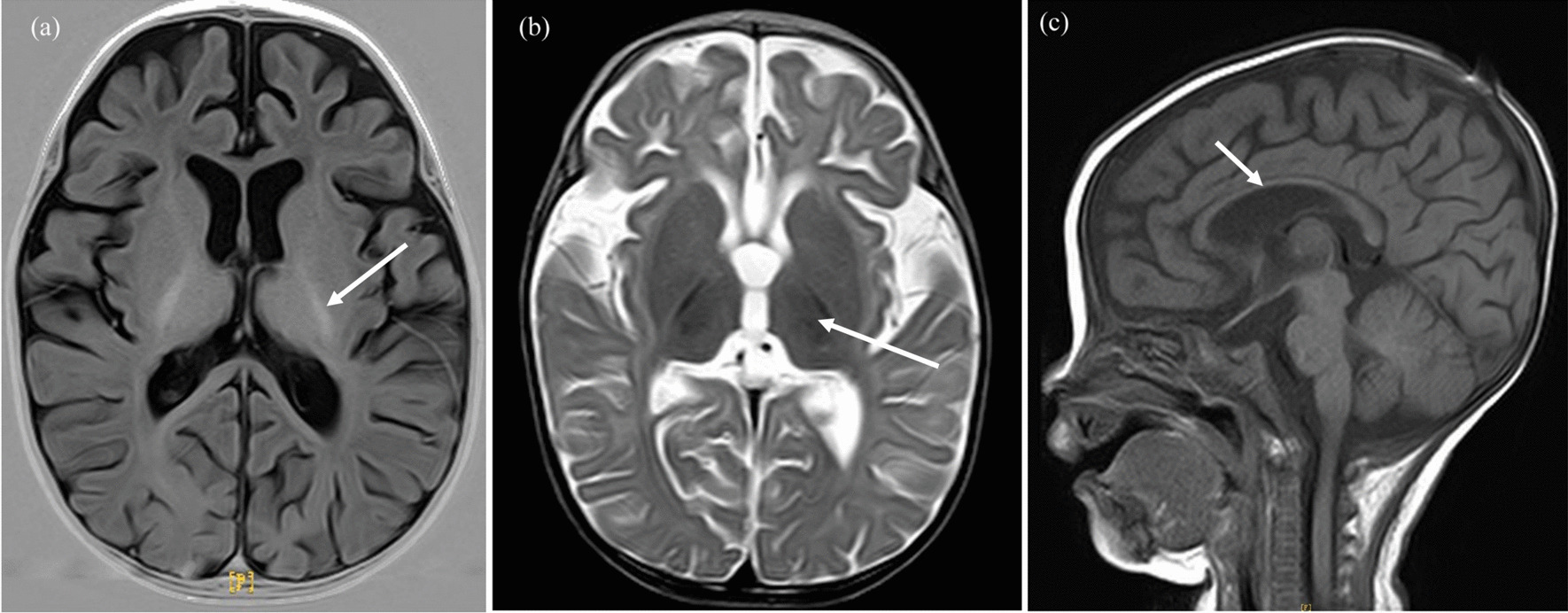
Brain magnetic resonance images (MRI) of the patient at seven months. T1‐weighted images (**a** and **c**) and T2‐weighted images (**b**). **a** The posterior limb of the internal capsule partly displays high intensity, suggesting myelination (arrows). **b** The posterior limb of the internal capsule displays low intensity, suggesting delayed myelination (arrows). **c** The gyri became bigger, and the sulci became deeper, while the hypoplastic corpus callosum showed hypointensity, suggesting progressive myelination (arrows)

When she was seven months old, she was admitted to the hospital with a fever as well as shortness of breath, abnormal vision, and drowsiness. The blood tests displayed anemia and liver dysfunction (Table 1). A physical examination revealed that the malnourished condition was uncorrected; the girl presented poor light reflection and poor visual acuity. Backward brain myelination was detected in the cranial MRI, whose mature myelination levels were equivalent to three months old infants (Fig. 3). Visual evoked potential (VEP) was abnormal, while auditory evoked potentials (AEP) were normal. The cardiac ultrasound demonstrated left ventricular enlargement with a small amount of pericardial effusion. Ophthalmological examination revealed retinal detachment, effusion, and necrosis. WES and trios whole-exon sequencing did not confirm the existence of gene variations strongly related to the disease characteristics. The elevated CMV VL was 94,200 copies/mL (Fig. 2). The refractory disseminated CMV disease was associated with organ involvement (pneumonitis, hepatitis, and retinitis).

Alternative therapies could not treat the immunodeficiency, and a panel of lymphocyte subsets demonstrated worsening T-cell lymphopenia (Table 2). Given the refractory CMV infection, we added foscarnet (FCV, 60 mg/kg, q.d.) with V-GCV to the therapy regimen without assay of the drug concentration or viral genetic alterations. GCV was injected into the vitreous cavity of the eyes, while there were 1.84E + 08 copies of CMV VL per milliliter of vitreous fluid. The anti-TB and antibiotic therapies were originally intended. During the 50-day treatment, the condition improved, including a decrease in VL and improved liver function and pneumonia. Her guardian requested discharge from the hospital without FCV.

**Fig. 4:**
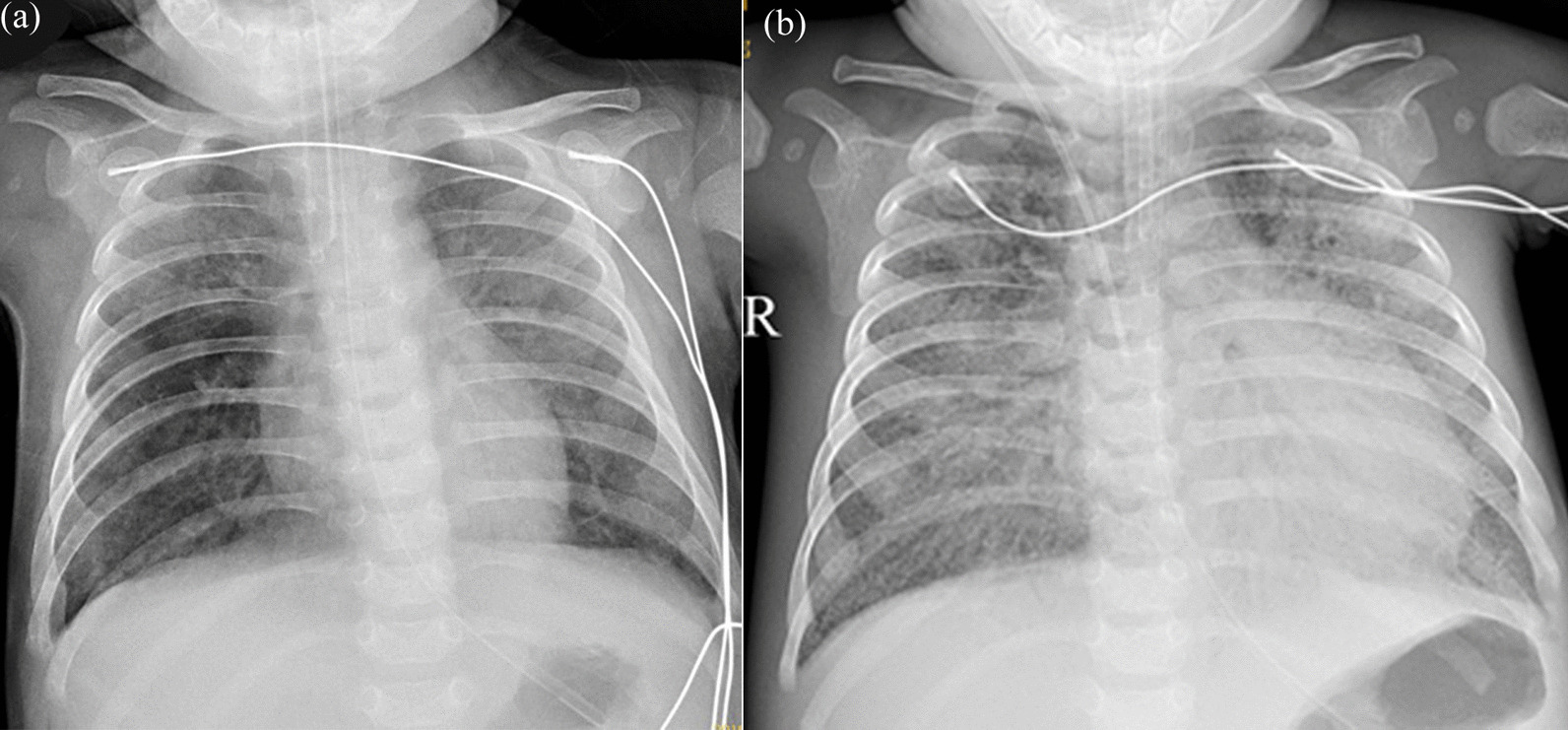
Chest X-ray images of the patient at three (**a**) and nine (**b**) months. Scattered, patchy shadows and interstitial changes

One month later, the girl was re-admitted to the hospital due to drowsiness and pneumonia. The patient presented severe global developmental delay with regression, and her weight dropped to 4.4 kg. Chest X-ray depicted severe pulmonary inflammation (Fig. 4). During the illness, she suffered from intermittent fever, septic shock (vital signs were as follows: Blood pressure of 78/54 mmHg, heart rate of 170 per minute, respiratory rate of 73 per minute and body temperature of 38.5 °C.). She was transferred to the ICU to get advanced life support measures. Her viral load has remained rising with deteriorating symptoms for the antiviral treatment with V-GCV, reaching 6.98E + 06 copies/ml (Fig. 2). The patient developed pneumothorax and subcutaneous emphysema, and died of progressive respiratory failure and septic shock on 10-month-old.

### Autopsy

**Fig. 5:**
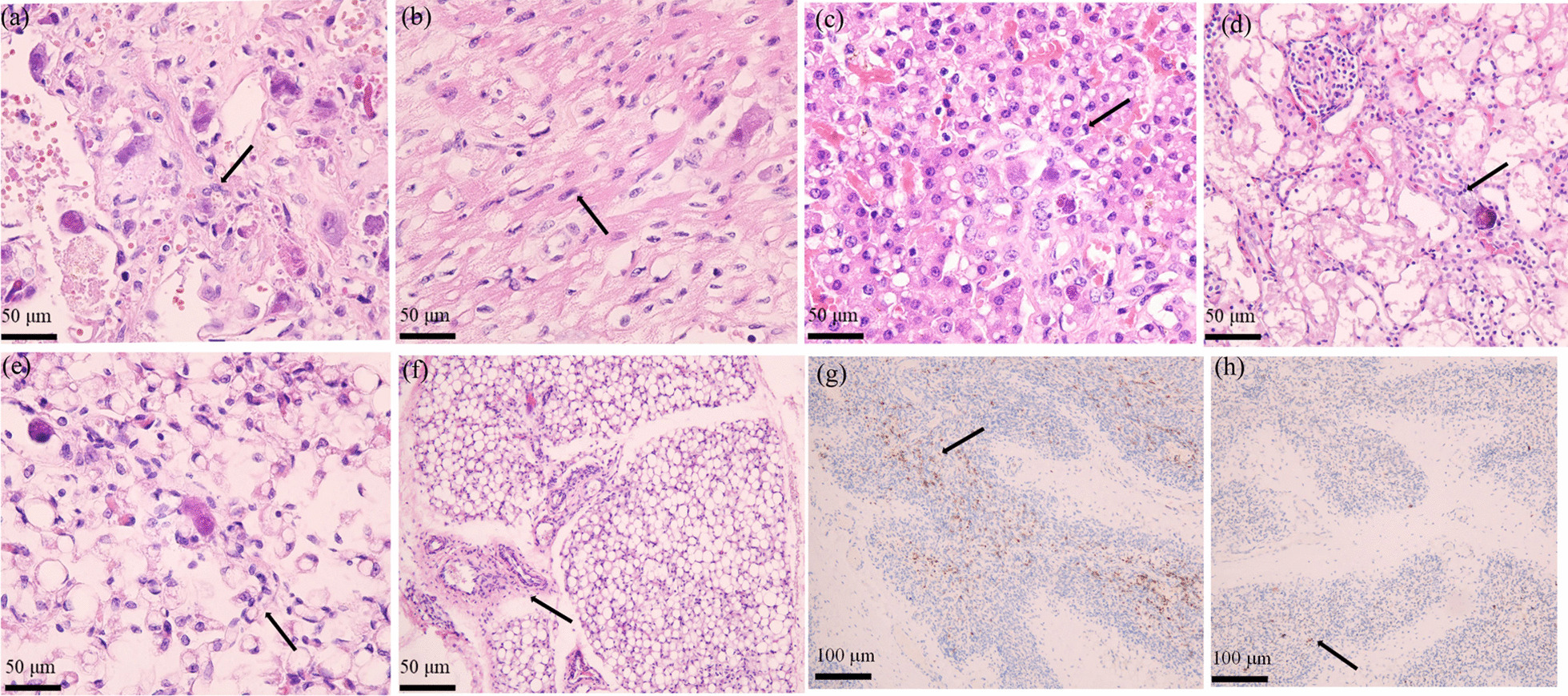
200 × Hematoxylin and eosin (H&E) stained appearance of CMV-infected tissues containing inclusion bodies like nucleolar cells (arrows): **a** lungs; **b** heart; **c** liver; **d** kidney; **e** thymus. **f** The number of thymic lobules is reduced, the poorly formed medullary disappears in the thymic corpuscle, and lymphocytes are significantly reduced, suggesting thymus dysplasia. **g** 100 × immunohistochemical (ICH) staining CD3 cells in the thymus. **g** 100 × immunohistochemical (ICH) staining of CD20, suggesting more positive CD3 T-cells than CD20 cells (yellow–brown)

An autopsy with organ involvement, including lungs, heart, liver, kidneys, and glands (pancreas, thyroid, and salivary gland tissues), was performed to confirm disseminated CMV infection. Containing inclusion bodies like nucleolar cells were found in the tissues (Fig. 5). H&E staining of the thymus revealed a reduction in the number of thymic lobules with cortical predominance, medulla showing a poorly formed disappearance of thymic corpuscle, all of which indicated thymus dysplasia. The immunohistochemistry depicted a larger amount of CD3 positive T-cells than that of CD20 positive B cells (Fig. 5).

## Discussion

CMV infection in immunocompetent individuals is usually asymptomatic, with few descriptions of dysfunction of one or more systems. Severe disseminated CMV infection typically develops in infants with primary maternal infections and during early gestation as well as in people with weakened host immune systems due to the reactivation of a latent virus [3]. At the moment, the features of reactivation and pathogenesis remain largely unclear [9]. Typical clinical features of active disseminated CMV disease include fever, leukopenia, thrombocytopenia, petechiae, pneumonitis, hepatitis, chorioretinitis, adrenalitis, and encephalitis. The universal newborn CMV screening that enables early diagnosis is recommended, whereas testing is now advised in cases of clinically suspected maternal primary CMV infection [10]. Acquired CMV infection can be life-threatening in immunocompromised patients, which can be divided into primary and secondary immunodeficiencies, which are caused by treatment for malignant neoplasms, HIV, and immunosuppressive therapy for solid organ or hematopoietic stem cell transplantation (HSCT). Further, impaired innate and adaptive immune responses contribute to the disease severity [3]. We summarize the characteristics of our case and compare them with different types of CMV infection in Table 3.

**Table 3:** Characteristics of this case compared with other Human Cytomegalovirus (CMV) Infections

CMV infection type	Population	Age at onset	Symptoms and signs	Treatment	Outcome
This case	Congenital thymic dysplasia	3 months old	Refractory CMV disseminated disease (retinitis, pneumonia, and hepatitis) and central nervous system involvement	GCV, V-GCV, and FOS	Death
Congenital cytomegalovirus( cCMV) Infection	Infants born to CMV-infected mothers	Pregnancy period	a. Asymptomatic; b. Asymptomatic during the newborn period with isolated SNHL; c. Symptomatic cCMV disease (approximately 10%), with manifestations including jaundice, petechiae, purpura, hepatosplenomegaly, microcephaly, intracerebral (typically periventricular) calcifications, SNHL, retinitis, and developmental delays	a. No antiviral therapy for asymptomatic; b. Not routinely receive antiviral treatment for mild symptomatic disease or with isolated SNHL (Uncertain); c. Six months of V-GCV therapy, only most severely affected symptomatic infants who are > 32 weeks gestation and less than 1 month of age; c. Monitoring of hearing and development	Death in 3% to 10% of symptomatic infants associated with primary maternal infections and occurs earlier during gestation; SNHL in approximately 10% of asymptomatic
Acquired CMV infection	Infants	Perinatal period	a. Asymptomatic and mild infections are the most common; b. Severe symptoms, end-organ disease (e.g., hepatitis, interstitial pneumonia, hematologic abnormalities including thrombocytopenia and leukopenia), and a viral sepsis syndrome	a. Mild cases should not receive antiviral treatment; b. Two weeks of GCV therapy, an additional 1 to 2 weeks can be considered if symptoms and signs have not resolved	The majority of them are curable, and few have a poor prognosis
	Secondary Immunodeficiencies ^a^	Highly variable	Severe symptoms are the same as above	a. Antiviral Pharmacotherapy (GCV, V-GCV, FOS, Cidofovir, Brincidofovir, Letermovir); b. Immune Globulin Intravenous (IGIV) or CMV-IGIV; c. CMV-Specific T-Cells Virus-specific T-cells	Vary with the age and immunocompetence of the host, life-threatening in resistant and refractory CMV infection
	Primary Immunodeficiencies	Usually during early childhood	a. Severe symptoms are the same as above; b. Haemophagocytic ymphohistiocytosis induces sustained systemic inflammatory responses and immune dysregulation, and autoimmune phenomena; persistence of infection with T-cell exhaustion		

*CMV* cytomegalovirus, *cCMV* congenital cytomegalovirus, *SNHL* sensorineural hearing loss, *GCV* ganciclovir, *V-GCV* valganciclovir, *FOS* foscarnet

a. Secondary Immune Deficiencies, including people receiving treatment for malignant neoplasms, people infected with human immunodeficiency virus (HIV), and people receiving immunosuppressive therapy for solid organ or hematopoietic stem cell transplantation

In this case, we could not determine cCMV infection or reactivation due to the absence of urine or saliva PCR in the first 21 days of life [1]. Despite receiving antiviral treatment with GCV and V-GCV, the VL was progressively related to a decrease in T-cells, which are recirculated into tissues where they perform antiviral effector functions, and by possible resistance owing to extended treatment. The virus were not genotyped due to lack of lab support that genotyping in UL97-mediated phosphorylation of GCV or other antiviral drug targets (e.g., UL54, UL97, UL56/89/51) [7], however this confirms that the refractory disseminated CMV infection was probably resistant to GCV, changing the VL by adjusting the antiviral therapy options. While prolonged therapeutic exposure may be a risk factor for resistance [6], long-term antiviral therapy aims to lessen or stop the progression of symptoms and late sequelae. Therefore, balancing the development of resistance and treatment continuation in clinical decision-making is challenging.

GCV, V-GCV, FCV, and Cidofovir antiviral medications are now approved for use in the treatment and/or prevention of infection. These drugs can prevent infection and disease in solid organ transplants, HSCT, and congenital infections. With the emergence of GCV-resistant CMV, other options, including FCV, Cidofovir, Maribavir, and Leflunomide, became available, protecting against infection with hyperimmune globulin [3]. VL monitoring and genotypic analysis are essential for detecting the early establishment of GCV resistance, while the usage and effectiveness of medicines should be evaluated against their possible side effects.

Importantly, the phenotype of T-cell lymphopenia did not allow any alternative therapy by WES and lymphocyte subset panel. Malnutrition can lead to adaptive immune deficiency [11]; however, both conditions exist simultaneously in our patients. In this case, the immunodeficiency of the patient was mainly manifested as T-cell lymphopenia, with evidence of the relative normal humoral immune response and pathology rather than malnutrition-related. Therefore, long-term malnutrition can exacerbate immune function deficiency. When CMV infection occurs, T-cells are crucial because CD4 + T-cells aid in co-stimulatory signals, while CD8 + T-cells are recirculated into tissues where they can perform antiviral effector activities and maintain long-term control of viral replication [9]. The main factor contributing to the poor prognosis, in this case, is thymic dysplasia, which prevents the host from having enough T-cells to generate efficient cellular autoimmunity [5]. The effectiveness of administering CMV-specific T-cells for treating and avoiding viral reactivation following HSCT has been demonstrated [12]. However, the uncertain defect phenotype and severe infections did not allow to match an appropriate stem cell protocol.

This case report describes the effects of arduous treatment and poor prognosis of CMV infection in a patient with primary immune deficiency of congenital thymic dysplasia or T-cell lymphopenia. Persistent elevations in DNA copies and aggravating complications (i.e., retinitis, pneumonia, hepatitis) can occur at the treatment stage. Therefore, vigilant monitoring of resistance and early etiology identification are required. Altogether, early aggressive early aggressive anti-CMV antiviral therapy in immunodeficiencies, VL monitoring, drug resistance and the role of T-cells in CMV infection are all important.

## Data Availability

All clinically relevant data have been made available within the manuscript.

## Abbreviations

CMV: Cytomegalovirus
PCR: Polymerase chain reaction
GCV: Ganciclovir
V-GCV: Valganciclovir
FCV: Foscarnet
WES: Whole exmore sequencing
cCMV: Congenital cytomegalovirus
SNHL: Sensorineural hearing loss
ICU: Intensive care unit
TB: Tuberculosis
VL: Viral load
